# Toxic small alarmone synthetase FaRel2 inhibits translation by pyrophosphorylating tRNA^Gly^ and tRNA^Thr^

**DOI:** 10.1126/sciadv.adr9624

**Published:** 2024-11-13

**Authors:** Tatsuaki Kurata, Masaki Takegawa, Takayuki Ohira, Egor A. Syroegin, Gemma C. Atkinson, Marcus J.O. Johansson, Yury S. Polikanov, Abel Garcia-Pino, Tsutomu Suzuki, Vasili Hauryliuk

**Affiliations:** ^1^ Department of Experimental Medical Science, Lund University, Lund, Sweden.; ^2^ RNA Systems Biochemistry Laboratory, RIKEN Cluster for Pioneering Research, 2-1 Hirosawa, Wako, Saitama 351-0198, Japan.; ^3^ Department of Chemistry and Biotechnology, Graduate School of Engineering, University of Tokyo, Bunkyo-ku, Tokyo 113-8656.; ^4^ Department of Biological Sciences, University of Illinois at Chicago, Chicago, IL 60607, USA.; ^5^ Department of Pharmaceutical Sciences, University of Illinois at Chicago, Chicago, IL 60607, USA.; ^6^ Center for Biomolecular Sciences, University of Illinois at Chicago, Chicago, IL 60607, USA.; ^7^ Cellular and Molecular Microbiology, Faculté des Sciences, Université libre de Bruxelles (ULB), Boulevard du Triomphe, Building BC, (1C4 203), 1050 Brussels, Belgium.; ^8^ WELBIO, Avenue Hippocrate 75, 1200 Brussels, Belgium.; ^9^ University of Tartu, Institute of Technology, Tartu, Estonia.

## Abstract

Translation-targeting toxic small alarmone synthetases (toxSAS) are effectors of bacterial toxin-antitoxin systems that pyrophosphorylate the 3′-CCA end of transfer RNA (tRNA) to prevent aminoacylation. toxSAS are implicated in antiphage immunity: Phage detection triggers the toxSAS activity to shut down viral production. We show that the toxSAS FaRel2 inspects the tRNA acceptor stem to specifically select tRNA^Gly^ and tRNA^Thr^. The first, second, fourth, and fifth base pairs of the stem act as the specificity determinants. We show that the toxSASs PhRel2 and CapRel^SJ46^ differ in tRNA specificity from FaRel2 and rationalize this through structural modeling: While the universal 3′-CCA end slots into a highly conserved CCA recognition groove, the acceptor stem recognition region is variable across toxSAS diversity. As phages use tRNA isoacceptors to overcome tRNA-targeting defenses, we hypothesize that highly evolvable modular tRNA recognition allows for the escape of viral countermeasures through tRNA substrate specificity switching.

## Teaser

The toxSAS FaRel2 inspects four base pairs of the tRNA acceptor stem to specifically select tRNA^Gly^ and tRNA^Thr^.

## INTRODUCTION

Toxin-antitoxin (TA) systems are ubiquitous prokaryotic regulatory systems. When active, the toxin abolishes bacterial growth, and its toxicity can be efficiently countered by the antitoxin, which can be either RNA- or protein-based (*1*). The most common type of TA is type II, where the protein toxin is neutralized by a protein antitoxin through formation of a tight complex. While multiple biological functions have been attributed to TAs, in recent years, it has become clear that many are antiphage defense systems that act through abortive infection mechanisms (*2*). TA effectors use numerous mechanisms of toxicity, often compromising translation (*3*). As an essential component of the translational machinery, transfer RNAs (tRNAs) are targeted by many different TA toxin families (*4*). To compromise translation, tRNAs can be cleaved by PilT N terminus (PIN)–containing toxins such as VapC (*5*–*8*) and MazF (*9*) or enzymatically modified by nucleotidyltransferases such as MenT (*10*); toxic small alarmone synthetase (toxSAS) pyrophosphokinases such as FaRel2, CapRel, PhRel, and PhRel2 (*11*–*13*); or GCN5-related N-acetyl-transferase (GNAT) acetyltransferases such as TacT (*14*), AtaT (*15*–*17*), and ItaT (*18*).

The substrate specificity of tRNA-targeting TA toxins has been an active topic of research. tRNA-cleaving PIN toxins are typically highly specific, with different members of the same family targeting different tRNA species (*5*–*9*). Substrate specificity of GNAT acetyltransferase toxins that catalyze aminoacyl-tRNA acylation varies from narrow [as in the case of AtaT2 from *Escherichia coli* O157:H7 that exclusively targets Gly-tRNA^Gly^ (*17*) or *E. coli* ItaT that specifically targets Ile-tRNA^Ile^ (*18*)] to relatively broad (*E. coli* AtaT modifies Gly-tRNA^Gly^, Trp-tRNA^Trp^, Tyr-tRNA^Tyr^, Phe-tRNA^Phe^, and Met-tRNA_i_^fMet^ (*16*), and a similarly broad specificity was reported for *Salmonella enterica* Enteritidis and *S. enterica* Typhimurium TacTs (*19*)). The nucleotidyltransferase MenT is particularly specialized. This toxin preferentially adds pyrimidines to the 3′-CCA end of just one tRNA species, tRNA^Ser^ (*10*). Phages exploit the tRNA specificity of tRNA-targeting defenses by expressing tRNA isoacceptors that are not recognized as substrates, thus allowing translation to continue. This strategy has been observed in the case of T5 phages that resist Retron (*20*) and PARIS (*21*) defense systems.

toxSASs are effectors of a recently found group of type II TA systems. These toxins are members of RelA/SpoT homolog superfamily and carry a catalytic domain—toxSYNTH—related to the (pp)pGpp alarmone synthetase domain (*22*, *23*). toxSASs use two distinct mechanisms of growth inhibition. *Cellulomonas marina* FaRel and the *Pseudomonas aeruginosa* type VI effector Tas1 synthesize the toxic alarmone (pp)pApp, which, in turn, causes depletion of adenosine 5′-triphosphate (ATP) (*11*, *24*, *25*). FaRel2, CapRel, PhRel, and PhRel2 toxSAS subfamilies on the other hand act as specific inhibitors of protein synthesis, pyrophosphorylating the 3′-CCA end of deacylated tRNA with ATP serving as a pyrophosphate donor (*11*–*13*).

The focus of the current study is FaRel2 toxSAS from *Сoprobacillus* sp. D7, a bacterial species that was recently renamed in National Center for Biotechnology Information (NCBI) databases to *Thomasclavelia ramose*. The prefix preceding the “Rel” indicates the taxonomic distribution of the toxSAS; FaRel2 is found in Firmicutes and Actinobacteria, hence the “Fa” prefix (*25*). FaRel2 toxin forms a nontoxic, enzymatically inert hetero-tetrameric complex with its cognate antitoxin ATfaRel2 (*26*). Prophage-encoded translation-targeting toxSAS TAs provide narrow-spectrum defense against superinfecting lytic phages, as it was shown for fused TA CapRel^SJ46^ (*13*), and bipartite TA PhRel:ATphRel (gp29:gp30) (*27*). While *Сoprobacillus* sp. D7 FaRel2:ATfaRel2 fails to afford antiphage protection when expressed in *E. coli* and tested against the BASEL phage collection (fig. S1A) (*28*), this is likely due to the use of a highly heterologous experimental system.

In our initial report describing tRNA pyrophosphorylation by toxSASs, we observed that FaRel2 is tRNA substrate specific: In biochemical assays, the enzyme modifies *E. coli* tRNA^Phe^ more efficiently than tRNA_i_^fMet^ and is exceedingly inefficient in pyrophosphorylating tRNA^Val^ (*11*). All of the three tested tRNAs belong to type I as per Brennan-Sundaralingam classification (*29*). Type I tRNAs have a short variable loop, while type II tRNAs tRNA^Ser^, tRNA^Tyr^, and tRNA^Leu^ have a long (>10 nt) variable loop with a helical stem of three to seven base pairs. Structural modeling suggests that the acceptor stem contains the primary substrate specificity determinants recognized by FaRel2 (*11*). Our original docking model of FaRel2 complexed with *E. coli* tRNA^Phe^ suggested that the 3′-CCA end of tRNA is guided into the FaRel2 active site through multiple contacts with the tRNA acceptor stem (*11*). The acceptor stem recognition is mediated by a basic patch of FaRel2, with K28A and R29A substitutions strongly reducing toxicity (*11*). In the neutralized FaRel2_2_:ATfaRel2_2_ TA complex, tRNA substrate binding by FaRel2 is seemingly unaffected; instead, the antitoxin exerts its neutralizing activity by precluding the binding of the ATP substrate (*26*). However, the binding experiments were performed with only one tRNA species, *E. coli* tRNA_i_^fMet^, yielding a *K*_d_ (dissociation constant) of 0.5 μM for both free FaRel2 and its antitoxin-naturalized form (*26*). The full in vivo substrate specificity of FaRel2 is unknown, and it is similarly unclear whether the tRNA binding specificity of FaRel2 is affected by TA complex formation.

In this study, we establish the tRNA specificity of FaRel2. We demonstrate that the toxin preferentially binds and modifies type I tRNAs tRNA^Gly^ and tRNA^Thr^. Both the free FaRel2 and the neutralized TA complex display the same tRNA specificity. Through structural modeling using AlphaFold 3 (AF3) (*30*), RNA substrate mutagenesis, and biochemical assays, we establish that four nucleotide base pairs in the acceptor stem of the tRNA determine tRNA substrate selection by FaRel2. We show that two translation-targeting toxSASs, *Bacillus subtilis* la1a PhRel2 (*11*) and fused toxin-antitoxin CapRel^SJ46^ (*13*), differ in tRNA specificity from FaRel2. Structural predictions of toxSAS:tRNA complexes by AF3 combined with conservation analyses provide the structural rational for divergent tRNA specificity among toxSAS: While the universal 3′-CCA element is recognized by a conserved positively charged groove, the acceptor stem is recognized by a highly divergent site.

## RESULTS

### Both the FaRel2 toxin and the neutralized FaRel2:ATfaRel2 complex preferentially bind type I tRNAs

**Fig. 1.**
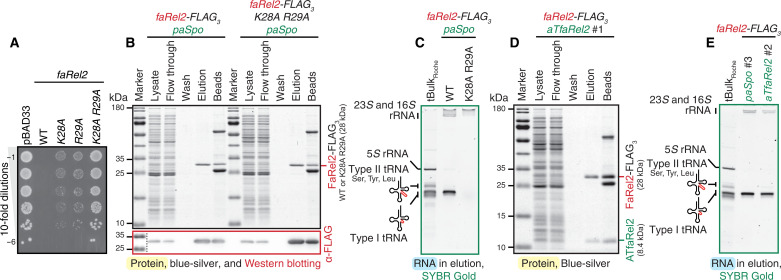
Immunoprecipitated FaRel2 associates with type I tRNAs. (**A**) Ten-fold dilutions of overnight cultures of *E. coli* strains transformed with pBAD33 vector or pBAD33 derivatives expressing either wild-type (WT) *Сoprobacillus* sp. D7 FaRel2 or alanine-substituted FaRel2 variants: K28A, R29A and the non-toxic double-substituted K28A R29A. (**B**) Purification of C-terminally FLAG_3_-tagged FaRel2 using anti-FLAG–conjugated beads. Samples were separated on SDS-PAGE and visualized by blue-silver staining and by Western blotting with anti-FLAG antibodies. To counter the toxicity of wild-type FaRel2, the toxSAS was coexpressed with PaSpo^SSU5^ SAH from *Salmonella* phage SSU5. (**C**) RNA coeluted with FaRel2-FLAG_3_ resolved on urea-PAGE and visualized by SYBR Gold staining. (**D**) Immunoprecipitation of FaRel2-FLAG_3_:ATfaRel2 for purification of co-IPed tRNA. (**E**) Comparison of the RNA samples coeluted with either FaRel2-FLAG_3_ or FaRel2-FLAG_3_:ATfaRel2 with a commercial preparation of *E. coli* small RNA fraction, tBulk_Roche_. Additional replicates are shown on fig. S2. All experiments were performed at least two times; representative images are shown.

To establish the tRNA specificity of FaRel2, we isolated toxin-bound tRNAs through FaRel2 immunoprecipitation and identified the enriched RNA species through modification-induced misincorporation tRNA sequencing (mim-tRNAseq) (*31*). For selective isolation of FaRel2 from BW25113 *E. coli* cells, we used a C-terminally FLAG_3_-tagged FaRel2 variant because, as we have shown previously, this engineered variant retains wild-type level of toxicity while being efficiently neutralized by the ATfaRel2 antitoxin (*25*). To improve the yield of FaRel2-FLAG_3_, we coexpressed it with the small alarmone hydrolase (SAH) PaSpo^SSU5^ from *Salmonella* phage SSU5. We previously showed that this SAH efficiently counteracts the toxic effects of FaRel2 by removing the tRNA modification installed by the toxin (*11*, *25*). Last, as a specificity control, we constructed a FaRel2 variant defective in tRNA binding. As we showed earlier, individual substitutions K28A and R29A that we predicted to disrupt the recognition of the acceptor stem of the tRNA strongly decrease the toxicity of FaRel2 (*11*). Here, we used a double-substituted K28A R29A FaRel2 protein variant that is nontoxic (Fig. 1A).

Immunoprecipitated FaRel2-FLAG_3_ and its K28A R29A derivative are highly homogeneous on the protein level (Fig. 1B). The RNA component of the FaRel2-FLAG_3_ sample is dominated by tRNA, with minor contamination by ribosomal RNA (rRNA); as expected, no tRNA band is detectable in the case of the K28A R29A variant (Fig. 1C). Sucrose gradient centrifugation and immunoblotting show that FaRel2 does not stably associate with 70*S* ribosomal complexes, further supporting the notion that the coimmunoprecipitated (co-IPed) tRNAs are directly bound to the toxin (fig. S1B). Comparison with the total small RNA sample from *E. coli* revealed that FaRel2-FLAG_3_ specifically coprecipitates with type I, but not type II, tRNA species. Note that in the following experiments, we used both a commercial product from Roche, tBulk_Roche_ (used as electrophoresis marker and in enzymatic assays), and our own tRNA preparations, designated simply as tBulk (used in enzymatic assays and as a control for mim-tRNAseq, see below).

After establishing the specificity of our pulldown procedure, we immunoprecipitated two types of samples for mim-tRNAseq: (i) FaRel2-FLAG_3_ coexpressed with PaSpo^SSU5^ SAH and (ii) FaRel2-FLAG_3_ coexpressed with ATfaRel2 antitoxin. The latter preparation contained substochiometric amounts of the antitoxin (Fig. 1D). Same as FaRel2-FLAG_3_, FaRel2-FLAG_3_:ATfaRel2 coprecipitated with type I tRNAs (Fig. 1E). Last, we repurified the tRNA fractions from rRNA contamination through sizing on a urea–polyacrylamide gel electrophoresis (PAGE) gel and used the resultant samples for mim-tRNAseq library preparation (≥1 μg of tRNA per sample; replicates and controls are shown in fig. S2).

### FaRel2 and FaRel2:ATfaRel2 preferentially bind tRNA^Gly^ and tRNA^Thr^

After deacylation of tBulk and immunoprecipitated tRNA samples, DNA adapters were ligated to tRNA CCA 3′ ends, the ligated RNA-DNA products gel-purified, subjected to reverse transcription, and resolved on urea-PAGE. The cDNA ran as two bands, one being a full-length product and the other being an aberrant product caused by reverse transcription stop (RT-stop) due to tRNA modifications (fig. S3). Both cDNA species were gel-purified, circularized, and polymerase chain reaction (PCR)–amplified. The resultant cDNA library was gel-purified once again before Illumina sequencing. The reads were processed and aligned to noncoding *E. coli* RNA genomic sequences. Taking advantage of 17-nt random Unique Molecular Identifier (UMI) D3N14 barcodes at the 5′ end of the RT primers, duplicated reads were removed after genome mapping, and the remaining reads were counted for individual tRNA genes (table S1).

**Fig. 2.**
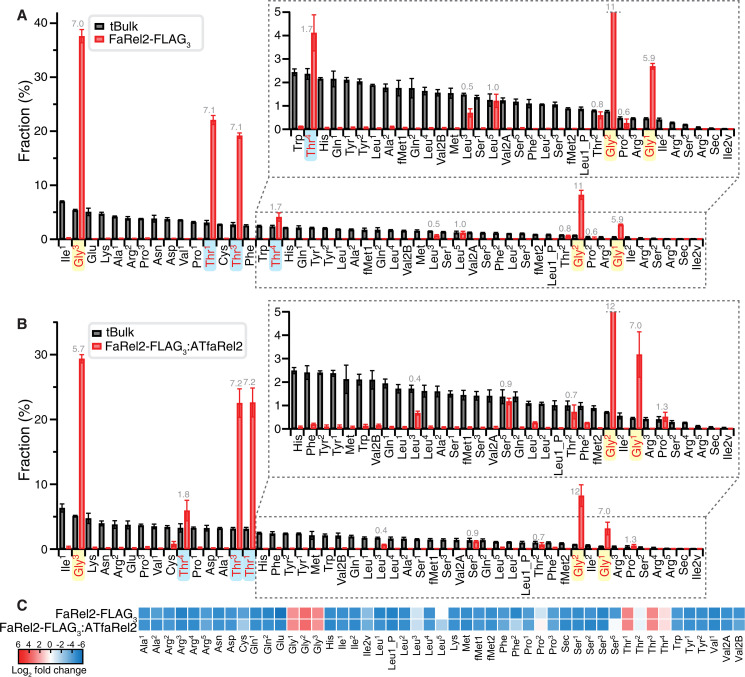
tRNA^Gly^ and tRNA^Thr^ are specifically bound by FaRel2 and FaRel2:ATfaRel2. (**A** and **B**) tRNA species abundance in (A) FaRel2-FLAG_3_- or (B) FaRel2-FLAG_3_:ATfaRel2–co-IPed RNA fraction and *E. coli* tBulk was quantified by mim-tRNAseq (*31*). Fold change of the relative tRNA abundance (fraction in co-IPed pool versus tBulk) is shown as gray numbers. (**C**) Heatmap representation for log_2_ fold change in abundance of tRNA species in co-IPed tRNA samples relative to their abundances in tBulk. The experiments were performed at least three times; the data are shown as average ± SD.

tRNA pools that copurify with both FaRel2 and FaRel2:ATfaRel2 are dominated by type I tRNAs tRNA^Gly^ and tRNA^Thr^ (Fig. 2, A to C). In the case of tRNA^Gly^, the FaRel2- and FaRel2:ATfaRel2-coimmunoprecipitated samples were strongly enriched in tRNA^Gly1^ (six- and sevenfold enrichment, respectively), tRNA^Gly2^ (11- and 12-fold), and tRNA^Gly3^ (six- and sevenfold). Strong enrichment was observed for tRNA^Thr1^ and tRNA^Thr3^ (both sevenfold), as well as a modest enrichment for tRNA^Thr4^ (twofold). The fraction of the tRNA^Thr2^ remains largely unchanged, which is indicative of a higher affinity than the majority of tRNA species that were depleted during immunoprecipitation. Despite being more abundant in tBulk, tRNA^Thr4^ (twofold enrichment) is less dominant in the immunoprecipitated pool as compared to tRNA^Gly2^ (11-and 12-fold enrichment), suggesting more efficient “capture” of tRNA^Gly2^ by FaRel2 in the cell. Notably, the three tRNA species that were used for biochemical studies of FaRel2 earlier—tRNA^Phe^, tRNA_i_^fMet^, and tRNA^Val^ (*11*, *26*)—were virtually absent in the immunoprecipitated tRNA pool. This, however, does not mean that FaRel2 has no affinity to these tRNA species (it was earlier established that FaRel2 binds *E. coli* tRNA_i_^fMet^ with a *K*_d_ of 0.5 μM (*26*)); rather, the weaker binders are outcompeted by the tighter binders.

### FaRel2 abrogates translation by preferentially targeting tRNA^Gly^ and tRNA^Thr^

**Fig. 3.**
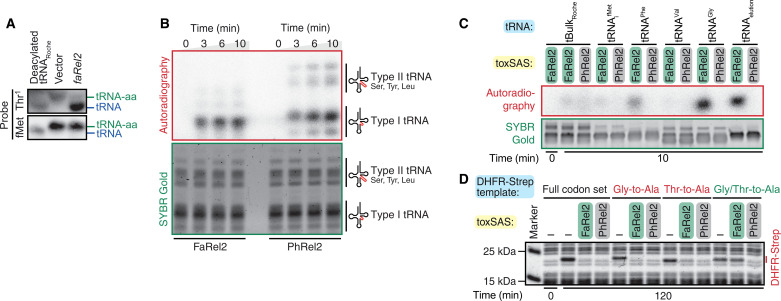
FaRel2 inhibits translation by specifically pyrophosphorylating tRNA^Gly^ and tRNA^Thr^. (**A**) Total RNA prepared from cells either expressing FaRel2 or transformed with an empty vector plasmid was resolved on acidic urea-PAGE and probed against tRNA^Thr1^ or tRNA_i_^fMet^. (**B** and **C**) tRNA pyrophosphorylation by FaRel2 or *B. subtilis* la1a PhRel2 assayed using ^32^P-labeled ATP. (B) FaRel2 specifically labels the type I tRNAs from *E. coli* total tRNA, tBulk_Roche_. A total of 50 nM toxSAS-FLAG_3_ was reacted with 5 μM tBulk_Roche_ at 37°C for increasing periods of time. tBulk_Roche_ stands for commercial preparation of *E. coli* small RNA fraction. (C) RNA^Gly^ and FaRel2-FLAG_3_–co-IPed tRNA fractions are more efficiently modified by FaRel2 as compared to tBulk_Roche_ and individual *E. coli* tRNAs tRNA_i_^fMet^, tRNA^Phe^, and tRNA^Val^. A total of 50 nM toxSAS-FLAG_3_ was reacted with 0.4 μM of tRNA preparations at 37°C for 10 min. (**D**) Reporter expression assays in cell-free protein synthesis system. Addition of FaRel2 abrogates production of Strep-tagged DHFR reporter proteins, which harbors full codon set or in which all Gly or Thr codons were substituted to Ala (Gly-to-Ala or Thr-to-Ala), but not the mutant variant in which all Gly and Thr codons were converted to Ala (Gly/Thr-to-Ala). Addition of *B. subtilis* la1a PhRel2 abrogates the expression of all reporters equally. All experiments were performed at least two times; representative gels are shown.

While the immunoprecipitation-based assays are informative in establishing the binding specificity of FaRel2, they do not necessarily reflect the enzymatic substrate preferences of the toxin. To address this question, we first assessed the effects of FaRel2 expression on tRNA_i_^fMet^ and tRNA^Thr1^ charging levels via Northern blotting assays. Consistent with the inferred substrate specificity, the expression of FaRel2 resulted in the disappearance of the acylated tRNA^Thr1^ without affecting the charging levels of tRNA_i_^fMet^ (Fig. 3A).

Next, we performed an in vitro tRNA pyrophosphorylation assay with FaRel2 using *E. coli* tBulk_Roche_ and ^32^P-ATP as substrates (*11*). In good agreement with the immunoprecipitation experiments, most of the ^32^P signal comes from type I tRNA species (Fig. 3B). As a specificity control, we performed the labeling assay with another translation-targeting toxSAS, *B. subtilis* la1a PhRel2 (*11*). In the case of PhRel2, the labeling pattern is different (Fig. 3B). Both type I and type II tRNAs are modified, and in the case of type I tRNA, both low and high-molecular weight species are labeled. When tested under same conditions, FaRel2 and PhRel2 were similarly efficient.

To probe the enzymatic specificity of FaRel2 further, we then performed pyrophosphorylation assays with (i) tBulk; (ii) native *E. coli* tRNA_i_^fMet^, tRNA^Phe^, tRNA^Val^, and tRNA^Gly^; or (iii) tRNA^Gly^- and tRNA^Thr^-enriched tRNA fractions isolated through anti-FLAG_3_ immunoprecipitation of FaRel2-FLAG_3_. Same as in the previous experiment, we used PhRel2 for comparison, and to increase the selectivity of tRNA modification, the tRNA substrate was used at lower concentration as compared to the previous experiment (0.4 μM versus 5 μM). tRNA co-IPed with either FaRel2-FLAG_3_ or FaRel2-FLAG_3_:ATfaRel2 was modified by FaRel2 more efficiently than either tBulk or tRNA_i_^fMet^; tRNA^Gly^ was modified as efficiently as the co-IPed tRNA (Fig. 3C and fig. S4). In the case of PhRel2, none of the tested tRNA species and fractions were modified efficiently (Fig. 3C), suggesting that tRNA_i_^fMet^, tRNA^Phe^, tRNA^Val^, tRNA^Gly^, and tRNA^Thr^ are not modified by PhRel2 efficiently.

Together, our results establish that (i) the preferential binding of tRNA^Gly^ and tRNA^Thr^ by FaRel2 is, indeed, reflective of the toxin’s enzymatic preferences, and (ii) the substrate preferences are toxSAS specific, as PhRel2 has a different specificity. We reasoned that, given FaRel2’s strong preference of these two specific tRNA species, expression of proteins that do not contain glycine or threonine should be largely insensitive to FaRel2. To test this, we used the reconstituted cell-free protein synthesis system from *E. coli* components [protein synthesis using recombinant elements (PURE)] (*32*). As we showed earlier, FaRel2 efficiently inhibits production of dihydrofolate reductase (DHFR) in the PURE system (*11*). We constructed four variants of the Strep-tagged DHFR reporter. The first variant was designed to contain the full set of 61 sense codons (designated as full codon set). The second and third variants were based on the full set reporter, but all of either glycine or threonine-encoding codons are substituted for alanine (designated as Gly-to-Ala and Thr-to-Ala, respectively). Last, we constructed a variant in which both glycine- and threonine-encoding codons are substituted for alanine, GT-to-A. All reporters described above were tested with FaRel2, PhRel2, and the fused toxin-antitoxin CapRel^SJ46^ (*13*). The latter enzyme is inactive unless triggered by the SECΦ27 phage major capsid protein Gp57 (*13*). Directly supporting our predictions, while the expression of the full codon set DHFR reporter and its Gly-to-Ala and Thr-to-Ala variants is readily abrogated by FaRel2, the expression of the Gly/Thr-to-Ala variant is insensitive to FaRel2 (Fig. 3E). Both PhRel2- (Fig. 3E) and Gp57-triggered CapRel^SJ46^ (fig. S5) efficiently inhibit the synthesis of all reporters, suggesting that these toxSAS toxins display a tRNA substrate specificity that is different from that of FaRel2.

### toxSASs combine a conserved CCA-recognizing groove with divergent substrate specificity regions

**Fig. 4.**
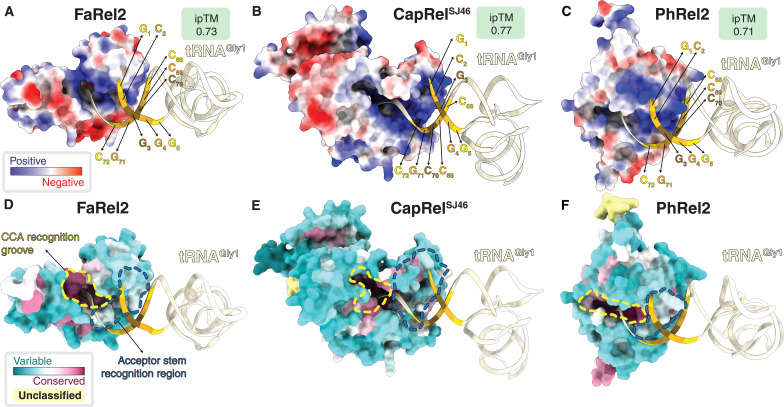
Conservation and diversification of tRNA recognition by toxSAS. (**A** to **C**) AF3-generated models of tRNA^Gly1^-bound *Сoprobacillus* sp. D7 FaRel2, *B. subtilis* la1a PhRel2, and CapRel^SJ46^ colored by the surface charge. (**D** to **F**) Same toxSAS:tRNA^Gly1^ complexes as on (A) to (C), but colored by amino acid conservation as computed using ConSurf (*33*).

To gain a structural insight into toxSAS tRNA selectivity, we predicted the structures of tRNA^Gly1^-bound FaRel2, PhRel2 and CapRel^SJ46^ in the ATP-liganded state using AF3 (*30*). We plotted charge distribution (Fig. 4, A to C) and conservation as calculated by ConSurf (Fig. 4, D to F) (*33*) on the protein structures. As we do not know which tRNA species are preferred by PhRel2 and CapRel^SJ46^, to simplify comparison, the same tRNA^Gly1^ was used for all predictions. The AF3-generated model of FaRel2:tRNA^Gly1^ is consistent with both the crystal structure of FaRel2 (*26*) and the FaRel2:tRNA docking model generated using HADDOCK (*34*) and validated through mutagenesis (*11*). Mutational analysis supports the AF3-generated models of tRNA^Gly1^-bound PhRel2 and CapRel^SJ46^ (fig. S6).

The predicted structures universally place the tRNA acceptor stem and the 3′-CCA to be recognized by a positively charged surface of the toxSAS (Fig. 4, A to C). The 3′-CCA is slotted into a deep and highly basic CCA recognition groove that extends to the toxSYNTH active site, while the first five base pairs of the acceptor stem interact with a shallower but similarly basic acceptor stem recognition region. While the CCA recognition groove is conserved among toxSASs, the acceptor stem recognition region is divergent, thus explaining the different tRNA substrate specificity among FaRel2, PhRel2, and CapRel^SJ46^ (Fig. 4, D to F).

### G_1_-C_72_, C_2_-G_71_, G_4_-C_69_, and G_5_-C_68_ serve as positive determinants for selection of tRNA^Gly^ and tRNA^Thr^ by FaRel2

**Fig. 5.**
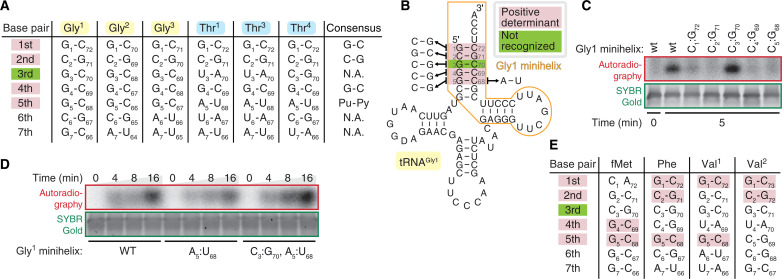
Molecular determinants defining tRNA selection by FaRel2. (**A**) Acceptor stem sequences for *E. coli* tRNA^Gly^ and tRNA^Thr^ isoacceptors. (**B**) *E. coli* tRNA^Gly1^ secondary structure and the tested base pair swapping mutations. The minihelix part is outlined with an orange line. Base pair swapping mutations are indicated by arrowheads. Pink background shows the base pairs where swapping mutation abrogates pyrophosphorylation of mini helix by FaRel2, and green background indicates the base pair where the swapping mutation did not decrease the pyrophosphorylation. (**C**) Pyrophosphorylation of tRNA^Gly1^-mimicking RNA minihelix by FaRel2 assayed with ^32^P-labeled ATP. Base pairs at positions 1-72, 2-71, 4-69, and 5-68 but that at 3-70 are crucial for substrate recognition by FaRel2. A total of 5 nM FaRel2-FLAG_3_ was reacted with 5 μM tRNA minihelix at 37°C for 5 min. The experiments were performed at least three times, and representative gels are shown. (**D**) Kinetic analysis of FaRel2-medited modification of wild type and A_5_:U_68_ and A_5_:U_68_ C_3_:G_70_ variants of tRNA^Gly1^-mimicking RNA minihelix. The experiments were performed analogously to (C). (**E**) Acceptor stem sequences for *E. coli* tRNA_i_^fMet^, tRNA^Phe^, tRNA^Val1^, and tRNA^Val2^.

Structural modeling suggests that FaRel2 contacts the first five base pairs of the acceptor stem of the tRNA (Fig. 4). This suggests that the substrate specificity should be encoded in this region of the tRNA molecule. Sequence comparison among tRNA^Gly1^, tRNA^Gly2^, tRNA^Gly3^, tRNA^Thr1^, tRNA^Thr3^, and tRNA^Thr4^ suggests that G_1_-C_72_, C_2_-G_71_, G_4_-C_69_, and Pu_5_-Py_68_ of the acceptor stem could serve as positive determinants for the recognition by FaRel2 because these nucleotides are conserved among the tRNA species that are preferentially recognized by the toxin (Fig. 5A). The third base pair is unconserved (Fig. 5A) and, therefore, is unlikely to be specifically recognized by FaRel2. While our analyses of FaRel2 tRNA specificity were performed in an *E. coli* surrogate host, tRNA^Gly^, tRNA^Thr^, and tRNA^Leu^ of the *Сoprobacillus* native host do contain all the predicted acceptor stems determinants (table S2).

To test the functional importance of these candidate positive specificity determinants, we used synthetic minihelix RNA oligonucleotides mimicking tRNA^Gly1^, a wild-type version and a series of base-flipping variants targeting the determinants such as G_1_-C_72_ to C_1_-G_72_ (Fig. 5B). Using native *E. coli* tRNA^Gly^ as a positive control, we have validated that our tRNA^Gly1^-mimicking RNA minihelix is efficiently modified by FaRel2, thus establishing the validity of our experimental system (fig. S7A). Base flipping of either of the candidate determinants—but not of the C_3_-G_70_ pair that is not conserved among the substrate tRNA species—compromised minihelix modification by FaRel2 (Fig. 5C). These results are consistent with the AF3-generated FaRel2:tRNA^Gly1^ model suggesting the lack of strong contacts between the C_3_-G_70_ base pair and the specificity region of FaRel2 (Fig. 4A). Our kinetically resolved experiments failed to detect any deleterious effect of substitutions of the C_3_-G_70_ pair, further supporting the notion that the base moieties of these nucleotides are not inspected by FaRel2 (fig. S7, B and C). While the substitutions in G_1_-C_72_ decreased the efficiency of modification markedly, this mutant was pyrophosphorylated more efficiently than those targeting C_2_-G_71_, G_4_-C_69_, and G_5_-C_68_, suggesting its relatively lower importance in substrate recognition (fig. S7C). The A_5_-U_68_ minihelix variant (this base pair is present in all the tRNA^Thr^ isoacceptors) was also efficiently modified, which is consistent with the conservation of this base pair in enriched tRNA species (Fig. 5D). The A_5_-U_68_ C_3_-G_70_ double substitution that mimics tRNA^Thr2^ and tRNA^Thr4^ did not change the modification efficiency (Fig. 5D) despite the relatively lower enrichment of these isoacceptors in co-IPed tRNA pools as compared to tRNA^Thr1^ and tRNA^Thr3^. Collectively, our results establish the critical role of G_1_-C_72_, C_2_-G_71_, G_4_-C_69_, and Pu_5_-Py_68_ (nucleotide positions as per tRNA^Gly1^) in tRNA substrate recognition by FaRel2.

## DISCUSSION

The tRNA acceptor stem is recognized by multiple tRNA-binding proteins, such as aminoacyl-tRNA synthetases (*35*), ProXp-ala (*36*) trans-editing protein that catalyzes the hydrolysis of mischarged Ala-tRNA^Pro^, peptidyl-tRNA hydrolase (*37*), elongation factors EF-Tu/eEF1A (*38*), and diverse toxins such as aminoacyl-tRNA acetylating AtaT (*16*), TacT (*39*), and ItaT (*40*); see recent review by Zhang (*41*). Here, we dissect one more class of acceptor stem–recognizing proteins: translation-targeting toxSAS toxins.

We show that the *Сoprobacillus* sp. D7 FaRel2 toxin specifically recognizes and pyrophosphorylates tRNA^Gly^ and tRNA^Thr^ and establish that base pairs G_1_-C_72_, C_2_-G_71_, G_4_-C_69_ and Pu_5_-Py_68_ of the tRNA acceptor stem (nucleotide positions are given as per tRNA^Gly1^) serve as specificity determinants that guide tRNA selection. These insights allow us to rationalize our previous biochemical results (*11*), specifically that *E. coli* tRNA^Phe^ is modified more efficiently than initiator tRNA_i_^fMet^, while tRNA^Val^ is an extremely poor substrate: While tRNA^Phe^ contains three determinants, both tRNA_i_^fMet^ and tRNA^Val^ contain only two. Specifically, tRNA^Phe^ has G_1_-C_72_, C_2_-G_71_ and G_5_-C_68_, tRNA_i_^fMet^ has G_4_-C_69_ and G_5_-C_68_, tRNA^Val^ isoacceptors have G_1_-C_72_ and either C_2_-G_71_ or G_5_-C_68_ (Fig. 5E). Last, we show that *B. subtilis* la1a PhRel2 and CapRel^SJ46^ have tRNA specificity different from that of FaRel2 and rationalize this observation by structural modeling and conservation analysis.

Our AF3-generated structural models suggest that tRNA selection by toxSAS is mediated by two recognition interfaces. While the universal CCA element is slotted into a highly conserved CCA recognition groove, the tRNA specificity determinants are inspected by the acceptor stem recognition region that is highly variable across the toxSAS diversity (Fig. 4). As we have shown earlier, even a single-strand 5′-CACCA-3′ RNA pentanucleotide can be modified by FaRel2 if used in a sufficiently high concentration (*11*). As this interaction would be mediated exclusively by the CCA recognition groove, it is to be expected that the affinity for this minimalistic substrate is low. The bipartite tRNA recognition mode of toxSAS is similar to that of aminoacyl-tRNA–modifying GNAT toxins such as AtaT (*16*) and TacT (*39*). Similar to the toxSAS, GNAT toxins interact with the CCA via a highly conserved active site patch while “reading” the major groove of the acceptor stem via a variable region (*16*, *42*).

Studies of GNAT toxins provide examples of other tRNA-targeting toxins that use tRNA^Gly^ as a substrate: *E. coli* AtaT2 exclusively selects Gly-tRNA^Gly^ (*17*), while *E. coli* AtaT modifies Gly-tRNA^Gly^ and Trp-tRNA^Trp^, Tyr-tRNA^Tyr^, Phe-tRNA^Phe^, and Met-tRNA_i_^fMet^ (*16*). It is tempting to speculate as to why tRNA^Gly^ would be especially well-suited as a substrate for toxins, and, in general, why toxSAS and GNAT toxins tend to be tRNA specific. It is possible that the high abundance of tRNA^Gly^ in the tRNA pool could be a contributing factor. As the toxin would undergo optimization during evolution, the specificity for a specific tRNA substrate (*k*_cat_/*K*_M_) would increase. Because in the case of toxSASs and GNATs, the tRNA regions that are recognized by the toxins differ among the tRNA species, this would drive the selection of tRNA-specific toxins. In other words, the specificity of tRNA-targeting toxins that recognize the acceptor stem is merely a by-product of their optimization toward efficient tRNA recognition. In the case of toxins involved in antiphage defense, this specificity can be then exploited by phages to counter tRNA-targeting antiviral defenses. Phages can overcome tRNA-targeting PARIS (*21*) and Retron (*20*) immunity systems by supplying alternative phage-encoded tRNA isoacceptors that are not recognized by the defense toxins. The evolutionary plasticity of the acceptor stem recognition region would allow tRNA specificity switching in diverse toxSAS, which would, in turn, enable escape of these counter-defense measures.

## MATERIALS AND METHODS

### Strain and plasmid construction

*E. coli* strains, plasmids, oligonucleotides, and synthetic DNA used in this study are listed in table S3. PCR-amplified DNA fragments were assembled by Gibson assembly kit (NEBuilder, NEB) as per the manufacturer’s protocol and introduced into DH5α *E. coli* strain. The assembled plasmids were amplified in the DH5α cells grown in LB (Lennox) liquid media, purified using the QIAprep Spin Miniprep Kit (QIAGEN), and sequenced.

### Protein expression and purification

C-terminally FLAG_3_-tagged *Coprobacillus* sp. D7 FaRel2 (FaRel2-FLAG_3_) was cloned into a pBAD33 derivative VHp678 under the control of arabinose-inducible P_BAD_ promotor. The toxin was overexpressed in *E. coli* BL21 DE3 cells cotransformed with the VHp701 plasmid encoding nontagged SAH ATfaRel antitoxin under the control of T7 promoter. Fresh transformants were used to inoculate an 800-ml culture [LB supplemented with kanamycin (50 μg/ml) and chloramphenicol (20 μg/ml)] to a final optical density at 600 (OD_600_) of 0.04. Bacteria were grown at 37°C until an OD_600_ of 0.3 when the antitoxin was pre-induced with 0.1 mM isopropyl-β-d-thiogalactopyranoside (IPTG) (final concentration) for 1 hour, after which the toxin was induced with 0.2% arabinose (final concentration) for an additional hour. The cells were collected by centrifugation [8000 rpm, 10 min at 4°C, JLA-10.500 rotor (Beckman Coulter)], dissolved in 4 ml of cell suspension buffer [20 mM Hepes:KOH (pH 7.5), 95 mM KCl, 5 mM NH_4_Cl, 0.5 mM CaCl_2_, 8 mM putrescine, 1 mM spermidine, 5 mM Mg(OAc)_2_, 1 mM dithiothreitol (DTT) and cOmplete protease inhibitor (Mini, EDTA-free from Roche)]. The cell suspension was divided to 1-ml aliquots, and 200 μl of prechilled zirconium beads (0.1 mm) was added to each of them. Cellular lysates were prepared by a FastPrep homogenizer (MP Biomedicals) (four 20-s pulses at speed 4.5 mp/s with chilling on ice for 2 min between the cycles) and clarified by centrifugation at 21,000*g* for 20 min at 4°C. The supernatant was carefully collected, avoiding the lipid layer and the cellular pellet.

The total protein (30 mg) as determined by Bradford assay of each sample was combined with 100 μl of ANTI-FLAG M2 Affinity Gel (Sigma-Aldrich) and mixed by rotation for 2 hours at 4°C. The mixture was loaded on a Micro Bio-Spin Chromatography Column (Bio-Rad), and flow through was collected. The gel in the column was washed five times with 1 ml of cell suspension buffer supplemented with 10% glycerol, and the fraction of the final wash was collected. Next, the gel was mixed for 20 min at 4°C on Multi Purpose Tube Rotator (Fisherbrand) together with 300 μl of elution buffer [cell suspension buffer additionally supplemented with 10% glycerol and Poly FLAG Peptide lyophilized powder (0.1 mg/ml; BioTools)]. The protein was eluted by briefly spinning down the column in a table-top centrifuge and collected in Eppendorf tube. After the elution step, the gel beads were resuspended with 1× sample buffer [50 mM tris:HCl (pH 6.8), 2% SDS, 0.01% bromophenol blue, 10% glycerol, 10 mM DTT, and 2% β-mercaptoethanol].

A total of 0.5 μl of cell lysate, 0.5 μl of flowthrough, 8 μl of wash, 8 μl of elution fraction, and 10 μl of gel suspension were resolved on 15% SDS-PAGE gel. The SDS-PAGE gel was treated with fixation solution (50% methanol and 2% phosphoric acid) for 5 min at room temperature, washed three times with water for 15 min at room temperature, and stained with “blue silver” solution [0.12% Brilliant Blue G250 (Sigma-Aldrich, 27815), 10% ammonium sulfate, 10% phosphoric acid, and 20% methanol] (*43*) overnight at room temperature. After the final 3-hour wash with water for at room temperature, the gel was imaged on Amersham ImageQuant 800 imaging system (Cytiva). The concentration of FaRel2-FLAG_3_ was estimated from SDS-PAGE gels by ImageJ (*44*) using pure ATfaRel2 as a standard.

### Toxicity assays

The assays were performed on LB medium (Lennox) plates (VWR). The *E. coli* BW25113 strain was transformed with pBAD33 derivatives expressing either wild-type *faRel2* or *faRel2* variants or an empty pBAD33 plasmid used as a vector control. The nucleotide sequence of the *faRel2* ORF was codon optimized for expression in *E. coli*. The cells were grown in liquid LB medium (BD) supplemented with chloramphenicol (20 μg/ml; AppliChem) and 0.2% glucose (repression conditions). Serial 10-fold dilutions were spotted (5 μl per spot) on solid LB plates containing chloramphenicol as well as either 0.2% glucose (repressive conditions) or 0.2% arabinose (induction conditions). Plates were scored after an overnight incubation at 37°C.

### Experimental phage infections

To assess the activity of FaRel2:ATfaRel2 TA system in phage defence, we performed efficiency of plating assays essentially as described previously (*45*). The experiments were performed using the BASEL coliphage collection (*28*) and a set of common laboratory phages. Briefly, *E. coli* BW25113 cotransformed with either VHp277 (pBAD33-*faRel2*) and VHp1199 (pMG25-*aTfaRel2*) or the empty pBAD33 and pMG25 vectors were grown overnight in LB medium supplemented with chloramphenicol (20 μg/ml) and ampicillin (100 μg/ml). The leaky *aTfaRel2* expression from VHp1199 is sufficient to neutralize the toxicity induced by *faRel2* expression (*26*). Bacterial lawns were prepared by mixing 0.75 OD_600_ units of cells with 10 ml of top agar (LB with 0.2% arabinose, 0.5% agar, 20 mM MgSO_4_, and 5 mM CaCl_2_) and overlaying this mixture on square LB-agar plates (1.5% agar) containing 0.2% arabinose. Phage stocks were 10-fold serially diluted in SM buffer [100 mM NaCl, 10 mM MgSO_4_, and 50 mM tris-HCl (pH 7.5)] and 2.5 μl of each of eight dilutions spotted on the solidified top agar plates. The formation of plaques was monitored after 6 and 24 hours of incubation at 37°C.

### Anti-FLAG_3_ immunoprecipitation of FLAG_3_-tagged FaRel2

C-terminally FLAG_3_-tagged *Coprobacillus* sp. D7 FaRel2 (FaRel2-FLAG_3_) was expressed from a pBAD33-derived plasmid VHp678 under the control of P_BAD_ promotor in *E. coli* BW25113 that was cotransformed with a pMG25 derivative encoding nontagged SAH PaSpo^SSU5^ from *Salmonella* phage SSU5 or ATfaRel2 antitoxin under the control of P_A1/O4/O3_ promoter. Fresh transformants were used to inoculate a 220-ml culture to a final OD_600_ of 0.05 in LB medium supplemented with ampicillin (100 μg/ml) and chloramphenicol (20 μg/ml). Expression of the ATfaRel2 antitoxin was preinduced with 150 μM IPTG. Expression of PaSpo^SSU5^ was not induced by IPTG as leaky expression from the P_A1/O4/O3_ promoter was sufficient to neutralize the FaRel2 toxicity. The cultures were grown at 37°C until an OD_600_ of 0.3, and then, the expression of FaRel2 was induced with 0.2% arabinose (final concentration). After 3 hours at 37°C, the culture was divided into two: 200 ml for pulldown and 20 ml for tBulk preparation; see the corresponding section of Materials and Methods. The cells were collected by centrifugation at 4000 rpm for 10 min at 4°C using S-4x universal rotor (Eppendorf). Cell pellets from the 200-ml culture were dissolved in cell suspension buffer [20 mM Hepes:KOH (pH 7.5), 95 mM KCl, 5 mM NH_4_Cl, 0.5 mM CaCl_2_, 8 mM putrescine, 1 mM spermidine, 5 mM Mg(OAc)_2_, 1 mM DTT, and MiniEDTA-free cOmplete protease inhibitor (Roche)] to final OD_600_ of 200. The cell suspension was divided to 1-ml aliquots, and 200 μl of pre-chilled zirconium and 0.1-mm beads were added to each aliquot. Cellular lysates were prepared using FastPrep homogenizer (MP Biomedicals) via four 20-s pulses at speed of 4.5 mp/s with chilling on ice for 2 min between the cycles. The lysates clarified by centrifugation at 21,000*g* for 20 min at 4°C, and the supernatant was carefully collected, avoiding the lipid layer and the cellular pellet.

Protein concentration in the supernatant was determined by Bradford assay, 5 mg of total protein per sample was combined with 100 μl of ANTI-FLAG M2 Affinity Gel (Sigma-Aldrich) and mixed on the end-to-end rotator for 2 hour at 4°C. The mixture was loaded on a Micro Bio-Spin Chromatography Column (Bio-Rad), and the flow through was collected and kept for further analysis. The column was washed five times with 1 ml of cell suspension buffer, and a fraction at final wash was collected. Using an end-to-end rotator, the gel was mixed on the column for 20 min at 4°C with 300 μl of cell suspension buffer supplemented with Poly FLAG Peptide (0.1 mg/ml; BioTools). The sample was eluted from the column by centrifugation and was collected in Eppendorf tube. In total, 900 μl of eluate was collected, and three technical replicates were performed for each biological replicate. After the elution step, the gel beads were suspended with 1× sample buffer [50 mM tris:HCl (pH 6.8), 2% SDS, 0.01% bromophenol blue, 10% glycerol, 10 mM DTT, and 2% β-mercaptoethanol] and kept for further analysis. A total of 0.5 μl of cell lysate, 0.5 μl of flow through, 8 μl of wash, 8 μl of elution fractions, and 10 μl of gel suspension were resolved on 12% SDS-PAGE gel. The SDS-PAGE gel was imaged. The FaRel2-FLAG_3_ concentration was estimated as described in the “Protein expression and purification” section.

The eluted sample (900 μl) was mixed with an equal volume of acidic phenol pH 4.3 and centrifugated [14,000 rpm for 20 min at 4°C in 5418 R Centrifuge equipped with FA-45-18-11 rotor (Eppendorf)]. The aqueous phase was collected, transferred to a fresh Eppendorf tube, and mixed with equal volume of chloroform, and the two phases were separated by centrifugation (14,000 rpm for 1 min at 4°C). The aqueous phase was again transferred to a fresh Eppendorf tube, mixed with 1:100 volume of glycogen (2 mg/ml), 3:50 volume of 5 M NaCl_2_, and 2.5 volume of 96% EtOH and kept at −20°C for overnight. Next, the tRNA sample was precipitated by centrifugation (14,000 rpm for 30 min at 4°C), and the pellet was washed with 200 μl of 70% EtOH. After air-drying at room temperature for 5 min, the pellet was dissolved in 10 μl of nuclease-free water.

By dephosphorylating the purified tRNA preparations, 9.5 μl of tRNA was combined with of T4 PNK ( 2 U/μl; NEB, M0201S) as well as N-terminally His_6_-TEV–tagged ATfaRel SAH (final concentration 1 μM) in a 20-μl reaction mixture (1× Polymix buffer with 5 mM Mg^2+^ final concentration additionally supplemented with 1 mM MnCl_2_ and 1 mM DTT). After a 10-min incubation at 37°C, the reaction was stopped by addition of 40 μl of RNA loading dye (98% formamide, 10 mM EDTA, 0.3% Bromophenol blue (BPB), and 0.3% xylene cyanol). A 60 μl of sample was resolved on 8 M urea-PAGE/tris-borate-EDTA (TBE) (8% acrylamide/bis-acrylamide = 19:1); 20 μl of the sample was loaded per lane. After staining the tRNA with SYBR Gold, the gel pieces containing tRNA were cut out and crushed in 1.5-ml Eppendorf tube. The crushed gel was combined with 1.2 ml of elution buffer (0.3 M NaOAc pH 5.2, 0.1% SDS, and 1 mM EDTA), and after shaking at 1500 rpm for 2 hours at 37°C, the supernatant was separated by passing the mixture through a 0.22-μm filter. The elution step was repeated one more time with fresh elution buffer, and collected tRNA elution samples were pooled. The pooled samples were mixed with 1:100 volume of glycogen (2 mg/ml) and 2.5 volume of 96% EtOH and then kept at −20°C overnight. tRNA solution was aliquoted in 1.5-ml tubes, and tRNAs were pelleted by centrifugation (14,000 rpm for 30 min at 4°C). The pellets were washed with 200 μl of 70% EtOH, and after air-drying pellet at room temperature for 5 min, the resultant tRNA preparations were kept at −80°C until use. To assess the tRNA yield and quality, the pellet was dissolved with nuclease-free water, and the concentration was measured spectrophotometrically assuming that one absorbance at 260 nm (*A*_260_) = 40 μg/ml.

### Preparation of *E. coli* tBulk

*E. coli* culture (20 ml) was prepared as described in the “Anti-FLAG_3_ pulldown and tRNA isolation” section. Cells were collected by centrifugation [4000 rpm, for 10 min at 4°C, S-4x universal rotor (Eppendorf)]. The cell pellets were dissolved in 400 μl of acidic cell suspension buffer [50 mM NaOAc and 10 mM Mg(OAc)_2_ (pH 5.2)] and mixed with 400 μl of acid phenol (pH 4.3) for 5 min at room temperature. The mixture was frozen in liquid nitrogen and thawed in water at room temperature. After one more round of this freeze and thaw, the sample was mixed by rotation for 2 hours at room temperature. Aqueous phase was separated by centrifugation [14,000 rpm for 10 min at room temperature in 5418 R Centrifuge equipped with FA-45-18-11 rotor (Eppendorf)], collected into fresh tube and mixed with 1 volume of TRI Reagent solution (Invitrogen, AM9738) and 0.1 volume of 1-bromo-3-chloropropane. The mixture was centrifugated (14,000 rpm for 10 min at room temperature), and aqueous phase was collected into fresh 1-ml tube. The aqueous phase was mixed with 1:20 volume of 3 M NaOAc and 1 volume of isopropanol and then kept at −20°C for 20 min. The total RNA was precipitated by centrifugation (14,000 rpm for 12 min at 4°C), and the supernatant was discarded. The RNA pellet was dissolved in 300 μl of nuclease-free water, mixed with 1:10 volume of 3 M NaOAc (pH 5.2) and 2.5 volume of 96% EtOH, and then kept at −20°C for 20 min. Total RNA was precipitated by centrifugation (14,000 rpm for 12 min at 4°C), the pellet was washed with 1 ml of 70% EtOH. After air-drying pellet at room temperature for 5 min, the pellet was dissolved with 7 μl of nuclease-free water. The total RNA solution was mixed with 14 μl of RNA loading dye (98% formamide, 10 mM EDTA, 0.3% BPB, and 0.3% xylene cyanol), and 21 μl of sample (in a lane) was resolved on 8 M urea-PAGE/TBE (8% acrylamide/bis-acrylamide = 19:1). After staining tRNA with SYBR Gold, the gel pieces containing tRNA bands were cut out and mashed in 1.5-ml tube. The mashed gel was mixed with 200 μl of 10 mM tris:HCl (pH 7.5) at 1500 rpm for 2 hours at 37°C, and the supernatant was collected with passing through 0.22-μm filter. This elution step was repeated one more time with fresh 10 mM tris:HCl (pH 7.5), and collected elution containing tBulk was pooled. The pooled elution was mixed with 1:100 volume of glycogen (2 mg/ml), 3:50 volume of 5 M NaOAc, and 2.5 volume of 96% EtOH and then kept at −20°C for overnight. tBulk was pelleted by centrifugation (14,000 rpm for 30 min at 4°C), the pellet was washed with 200 μl of 70% EtOH. After air-drying pellet at room temperature for 5 min, the pellet was dissolved in 10 μl of nuclease-free water, and the concentration was measured by the *A*_260_ value (one *A*_260_ = 40 μg/ml). Ten micrograms of tBulk was dephosphorylated and repurified as described in the “Anti-FLAG_3_ pulldown and tRNA isolation” section.

### Expression and purification of native glycine-specific tRNA^Gly^

The native tRNA was expressed and purified essentially as described previously (*46*) In brief, *E. coli* tRNA^Gly1^ (CCC anticodon) genomic sequence was assembled from five oligonucleotides, supplemented with flanking Eco RI and Pst I restriction sites (see table S3).

The assembled tRNA genomic sequence was then inserted into the Eco RI/Pst I–digested pBSTNAV vector (Addgene, USA). The final construct contained the tRNA under the control of the constitutive lpp promoter. The cells were grown overnight (16 hours) in LB media supplemented with ampicillin (100 μg/ml), and the biomass was collected by centrifugation. It was resuspended in 1 mM tris-HCl (pH 7.4) and 10 mM Mg(CH_3_COO)_2_ and lysed with 0.5 volumes of acidic phenol:chloroform mix of 5:1 (pH 4.5). The aqueous phase was precipitated and resuspended in 1 M NaCl. The solution, containing soluble RNAs, was precipitated again. To deacylate bulk tRNA, the pellet was resuspended in 200 mM tris-HCl (pH 9.0) and incubated for 2 hours at 37°C. The deacylated tRNA was ethanol precipitated and dissolved in monoQ buffer A [40 mM sodium phosphate buffer (pH 7.0)] and separated on the 8-ml MonoQ column (10/100, GE Healthcare) using a two-step linear gradient of buffer B (A with 1 M NaCl): 20 ml of 0 to 50% B followed by 280 ml of 50 to 100%. The tRNA^Gly1^-containing fractions were identified by analytical aminoacylation with [^14^C]-glycine using *Thermus thermophilus* GlyRS. Pooled fractions were precipitated and dissolved in C5 buffer A [20 mM NH_4_CH_3_COO (pH 5.5), 400 mM NaCl, 10 mM MgCl_2_, 1 mM EDTA, and 1% methanol]. Further reverse-phase chromatography on the C5 column (C5-5, 250 × 10 mM, Discovery BIO Wide Pore, Supelco) using 300 ml of 0 to 60% linear gradient of C5 buffer B (buffer A supplemented with 40% methanol) produced pure tRNA preparation with more than 95% glycine-charging activity.

### mim-tRNAseq tRNA sequencing and data analysis

mim-tRNAseq was performed as described previously (*31*) with minor modifications. Seven pmol of tBulk and FaRel2-bound tRNA prepared from *E. coli* cells coexpressing FaRel2-FLAG_3_ with either ATfaRel2 or PaSpo^SSU5^ was deacylated under 100 μl of 100 mM CHES-NaOH (pH 9.0) for 1 hour at 37°C and purified by Oligo Clean & Concentrator (Zymo Research). Before adapter ligation, DNA adapter was adenylated in a 30 μl of reaction mixture consisting of 6 μM DNA adapter, 5 μM Mth RNA ligase (NEB), 1× adenylation buffer (NEB), and 1 mM ATP and purified by Oligo Clean & Concentrator. The deacylated tRNA was ligated with the DNA adapter to the 3′-end in a 20 μl of reaction mixture consisting of 28 pmol of adenylated DNA adapter; 200 U T4 RNA ligase 2 truncated KQ (NEB); 1× T4 RNA ligase buffer (NEB); 25% polyethylene glycol, molecular weight 8000; and 10 U SUPERase·In (Thermo Fisher Scientific) at 22°C overnight. After ligation, the mixture was purified by Oligo Clean & Concentrator and resolved on a urea-PAGE in 1× TBE (7 M urea and 10% PAGE). The gel was stained with SYBR Gold (Thermo Fisher Scientific), and the gel image was acquired by a FAS-DIGI PRO (NIPPON Genetics). Visualized bands corresponding to adapter-ligated tRNAs were excised from the gel and eluted for 3 hours at 37°C with continuous mixing in 400 μl of elution buffer consisting of 400 mM NaOAc (pH 5.2), 0.1% SDS, and 1 mM EDTA-NaOH (pH 8.0). The elute was filtered by Ultrafree-MC (Merck) to remove the pieces of mashed gel and then subjected to ethanol precipitation with glycogen (20 μg/ml). For reverse transcription, adapter-ligated tRNA was mixed with 2.5 pmol of reverse transcription (RT) primer in a 11 μl of water, denatured at 82°C for 2 min, annealed at 25°C for 5 min, and then applied to reverse transcription at 42°C overnight in a 20 μl of reaction mixture consisting of 1× RT buffer [50 mM tris-HCl (pH 8.3), 75 mM KCl, and 3 mM MgCl_2_], 5 mM DTT, 5 mM deoxynucleotide triphosphates (dNTPs), 20 U of SUPERase·In, and 1 μl of TGIRT-III (InGex). Following reverse transcription, tRNA template was hydrolysed by alkaline treatment with 100 mM NaOH at 95°C for 2 min, and synthesized cDNA was purified by Oligo Clean & Concentrator, followed by gel purification as described above. Obtained cDNA was circularized at 60°C overnight in a 20 μl of reaction mixture consisting of 10 U with CircLigase ssDNA ligase (Lucigen), 1× reaction buffer (Lucigen), 2.5 mM MnCl_2_, 1 M betaine, and 50 μM ATP and incubated at 80°C for 10 min. One microliter of circularized cDNA was subjected to PCR in a 20 μl of reaction mixture composed of Phusion polymerase (20 U/ml; NEB), 1× Phusion GC buffer (NEB), 0.2 mM dNTPs, 3% dimethyl sulfoxide, 0.5 μM forward primer, and 0.5 μM reverse primer. The reaction was performed with initial denaturation at 98°C for 30 s, followed by 8 or 10 cycles of 98°C for 10 s, 60°C for 20 s, and 72°C for 5 s. PCR product was purified by AMPure XP (Beckman Coulter) and further purified by gel extraction with native-PAGE in 1× TBE (6% PAGE). Obtained cDNA libraries were purified by AMPure XP and quantified by Agilent 2100 Bioanalyzer system (Agilent). A total of 500 pmol of each cDNA library was mixed in a 30 μl of water and sequenced on an Illumina HiSeq X Ten platform (150 bp, pair end).

Raw Illumina sequencing reads were trimmed to remove both adapter and read 1 or 2 sequences using cutadapt 4.4 (default version). Only read 1 sequence data were used for subsequent processing. Following trimming, 17 nt of random UMI sequence (N14D3) attached to 5′ side of tRNA sequence was further removed from each read and written into the read name as a UMI tag by fastp v0.23.2. The reads with shorter than 15 nt, the quality score at 5′ and 3′ ends below 20 was discarded by Trimmomatic 0.39, and the filtered reads were then aligned to the sequences of *E. coli* noncoding RNAs including all tRNAs [obtained from NCBI database (*E. coli* BW25113 complete genome, GenBank CP009273.1] using bowtie 2-2.5.1-linux-x86_64 (*47*) with very sensitive local mode and -L 10. PCR duplicates were deduplicated on the basis of the UMI tag by UMICollapse. The mapped read numbers on each tRNA gene were counted by samtools-1.14. The *P* value was calculated by two-sided Student’s *t* test (*n* = 3). Statistics of the individual tRNA coverage (read counts) for mim-tRNAseq experiments are provided in table S1.

### tRNA Northern blotting

We used *E. coli* BW25113 strain transformed either with pBAD33 derivative expressing wild-type *faRel2* or empty pBAD33 as vector control. The cells were grown in liquid LB medium (BD) supplemented with chloramphenicol (20 μg/ml; AppliChem) and 0.2% glucose (to repress the FaRel2 expression). Fresh transformants were used to inoculate a 40-ml culture [LB supplemented with chloramphenicol (20 μg/ml)] to a final OD_600_ of 0.05. Bacteria were grown at 37°C until an OD_600_ of 0.3, and then, FaRel2 expression was induced for 30 min with 0.2% arabinose (final concentration). The cells from a 25-ml culture were collected by centrifugation [4000 rpm, 10 min, at 4°C in S-4x universal rotor (Eppendorf)] and resuspended in 0.5 ml of 3 M NaOAc (pH 4.5) supplemented with 10 mM EDTA. The cells mixed with 0.5 ml of acid phenol and 20 μl BCP (1-Bromo-3-chloropropane) and kept on ice for 15 min. After centrifugation [14,000 rpm for 20 min at 4°C in 5418 R Centrifuge equipped with FA-45-18-11 rotor (Eppendorf)], the aqueous phase was removed and mixed with an equal volume of isopropanol and kept at −20°C for an hour. Total RNA was precipitated by centrifugation (14,000 rpm for 20 min at 4°C), and the RNA pellet was washed with 1 ml of 70% ethanol. The RNA pellet was dissolved in 20 μl of 10 mM NaOAc (pH 4.5) supplemented with 1 mM EDTA, and the total RNA concentration was determined spectrophotometrically (one *A*_260_ = 40 μg/ml). The RNA sample was mixed with ≥5 volumes of acid urea-PAGE sample buffer (98% formamide, 10 mM EDTA, 0.3% BPB, and 0.3% xylene cyanol for nucleic acid staining), and 6 μg of RNA was resolved on 8 M urea-PAGE/100 mM NaOAc (pH 5.2) (6.5% acrylamide:bis-acrylamide in 19:1 ratio). RNA was transferred to Zeta-Probe Blotting Membranes (Bio-Rad) using Trans-Blot TurboTM Transfer System (Bio-Rad) and ultraviolet cross-linked on menbrane, and the membrane was prehybridized at 42°C for ≥3 hours in preheated Church buffer (0.25 mM Na_2_HPO_4_, 0.17% orthophosphoric acid, 1 mM EDTA, 0.01% bovine serum albumin, and 0.07% SDS). After discarding the used Church buffer, 13 nM ^32^P-labeled probe was hybridized in fresh Church buffer for ≥16 hours at 42°C. The membrane was washed three times with 0.1% SDS/6x saline-sodium citrate (SSC) at 42°C for 5 min and exposed on an imaging plate, and the plate was imaged by a FLA-3000 (Fujifilm).

### tRNA pyrophosphorylation assays

With the exception of the experiment shown in Fig. 3B, FaRel2-FLAG_3_–mediated tRNA modification was assayed in two enzymatic regimes: (i) low enzyme concentration (5 to 10 nM) combined with high substrate concentration (5 μM) and (ii) high enzyme concentration (50 to 100 nM) combined with low substrate concentration (0.4 μM). All experiments were performed in Hepes:Polymix buffer, 5 mM Mg^2+^ final concentration.

FaRel2-FLAG_3_ was reacted with either (i) co-IPed tRNA, (ii) tBulk purified from *E. coli* as described in the “Preparation of *E. coli* tBulk” section (see above), (iii) commercial tBulk from Roche (tBulk_Roche_), or (iv) *E. coli* tRNA_i_^fMet^, tRNA^Phe^, tRNA^Val^ (all from Chemical Block), and tRNA^Gly^ (purified as described in the “Expression and purification of native glycine-specific tRNA^Gly^” section, see above). Conditions for experiments presented on different figures are as follows: Fig. 3B: 50 nM toxSAS, 5 μM tRNA substrate, and reaction time of 3 to 10 min; Fig. 3C: 50 nM toxSAS, 0.4 μM tRNA substrate, and reaction time of 10 min; Fig. 5C: 5 nM toxSAS, 5 μM tRNA substrate, and reaction time of 5 min; Fig. 5D: 10 nM toxSAS, 5 μM tRNA substrate, and reaction time of 4 to 16 min; fig. S4: 100 nM toxSAS, 0.4 μM tRNA substrate, and reaction time of 10 min; fig. S7A: 5 nM toxSAS, 5 μM tRNA substrate, and reaction time of 5 min; fig. S7C: 10 nM toxSAS, 5 μM tRNA substrate, and reaction time of 4 to 16 min.

The reactions were started by the addition of 500 μM γ^32^P-ATP and carried out at 37°C for 0.25, 3, 5, or 10 min. To visualize pyrophosphorylated tRNA, the reaction sample was mixed in 2 volumes of RNA dye (98% formamide, 10 mM EDTA, 0.3% bromophenol blue, and 0.3% xylene cyanol), tRNA-denatured at 37°C for 10 min, and resolved on urea-PAGE in 1× TBE (8 M urea and 8% PAGE). The gel was stained with SYBR Gold (Life technologies, S11494) and imaged using Amersham ImageQuant 800. Next, the gel was exposed to an imaging plate overnight, and the imaging plate was imaged by a FLA-3000 (Fujifilm). In the case of kinetically resolved experiments, the signal intensities from the SYBR Gold staining and the phosphorimaging were quantified using ImageJ (*44*) software. The data were processed as follows: (i) The relative loading amounts of individual minihelix species were calculated as ratio of averaged signals from SYBR gold staining of individual loading replicates, (ii) individual phosphorimaging signal intensities were normalized to the relative loading amount of the minihelix species, (iii) efficiency of the pyrophosphoate labeling was calculated as percentile to the normalized intensity of wild type at 16 min (set to 100%), and (iv) the average and the SD were calculated using the data from three independent experiments.

### Cell-free translation assays

Experiments with PURExpress In Vitro Protein Synthesis Kit (NEB, E6800) were performed as per the manufacturer’s instructions using DHFR-Strep reporter plasmid (10 ng/μl) with the addition of RNase Inhibitor Murine (0.8 U/μl; NEB, M0314S). Purified FaRel2-FLAG_3_ (at 50 nM as final concentration) or PhRel2-FLAG_3_ (at 100 nM as final concentration) was used, and as a mock control, toxSASes was substituted for equal volume of Hepes:Polymix buffer. The total reaction volume was 6 μl per reaction for most of the experiments. After incubation at 37°C for the indicated time, the reaction mixture was mixed with ninefold volume of 2x sample buffer [100 mM tris:HCl (pH 6.8), 4% SDS, 0.02% bromophenol blue, 20% glycerol, 20 mM DTT, and 4% β-mercaptoethanol], and 5 μl of the mixture was resolved on 18% SDS-PAGE gel. In the case of CapRel^SJ46^, purified CapRel^SJ46^ protein was used at a final concentration of 250 nM, with *gp57*, an essential activator for CapRel^SJ46^, as template plasmid at 10 ng/μl. As a mock control, CapRel^SJ46^ was substituted for equal volume of Hepes:Polymix buffer. After a 10-min incubation at 37°C, a 1.34-μl aliquot of the reaction mixture was taken and quenched by addition of 13.66 μl of 2× sample buffer, and DHFR-Strep reporter plasmid solution (193 ng/μl) was added to the remaining reaction mixture at a final concentration of 20 ng/μl. After further incubation at 37°C for 1 hour, the reaction mixture was mixed with ninefold volume of 2× sample buffer and 5 μl of the mixture was resolved on 18% SDS-PAGE gel. The SDS-PAGE gel was fixed by incubating for 5 min at room temperature in 50% methanol solution supplemented with 2% phosphoric acid and then stained and detected as mentioned in protein expression and purification.

### Sucrose gradient fractionation and Western blotting

C-terminally FLAG_3_-tagged *Coprobacillus* sp. D7 FaRel2 variant (FaRel2-FLAG_3_ K28A R29A) was expressed from arabinose-inducible P*_BAD_* promotor on pBAD33-derivative VHp1093 in *E. coli* BW25113 cells cotransformed with the VHp847 plasmid encoding *Salmonella* phage SSU5 SAH PaSpo under the control of IPTG-inducible P*_A1/04/03_* promoter. Fresh transformants were used to inoculate a 100-ml culture [LB supplemented with ampicillin (100 μg/ml) and chloramphenicol (20 μg/ml)] to a final OD_600_ of 0.05. Bacteria were grown at 37°C until an OD_600_ of 0.3 without IPTG, and then FaRel2-FLAG_3_ K28A R29A was induced with 0.2% arabinose (final concentration) for an hour. The cells from 50-ml culture were collected by centrifugation [4000 rpm, 10 min, at 4°C in S-4x universal rotor (Eppendorf)], frozen in liquid nitrogen, and stored at −80°C until use. The cells were melted on ice and dissolved in 0.5 ml of Hepes:Polymix buffer [(*48*); 5 mM Mg^2+^ final concentration] supplemented with 1 mM phenylmethylsulfonyl fluoride, lysed using FastPrep homogenizer (MP Biomedicals) (four 20-s pulses at 4.0 m/s with chilling on ice for 2 min between the cycles), and clarified by centrifugation [14,000 rpm for 20 min in 5418 R Centrifuge equipped with FA-45-18-11 rotor (Eppendorf)]. Ten *A*_260_ units of the lysate were loaded onto 10 to 35% sucrose gradient in Hepes:Polymix buffer (pH 7.5) (5 mM Mg^2+^ final concentration) and resolved by ultracentrifugation at 36,000 rpm for 3 hours at 4°C with the fastest braking in Optima XPN-80 Ultracentrifuge equipped with SW-41Ti rotor (Beckman Coulter). Gradients were fractionated (0.5 ml per fraction) using Biocomp Gradient Station (BioComp Instruments) with *A*_260_ as a readout. For Western blotting, 0.5-ml fractions was supplemented with 1.5 ml of 96% ethanol and precipitated overnight at −20°C. After centrifugation at 14,000 rpm for 30 min at 4°C, the supernatants were discarded. and the samples were dried. The pellets were resuspended in 40 μl of 1x SDS loading buffer [50 mM tris-HCl (pH 6.8), 2% SDS (w/v), 0.01% bromophenol blue, 10% glycerol (w/v), and 2% β-mercaptoethanol], resolved on the 10% SDS PAGE, and transferred to nitrocellulose membrane (Trans-Blot Turbo Midi Nitrocellulose Transfer Pack, Bio-Rad; 0.2-μm pore size) with the use of a Trans-Blot Turbo Transfer Starter System (Bio-Rad). Membrane blocking was done for 1 hour in PBS-T (1× PBS and 0.05% Tween 20) with 5% (w/v) nonfat dry milk at room temperature. FaRel2 was detected using anti-FLAG M2 antibody (1:10,000 dilution; Sigma-Aldrich, F1804) primary, combined with goat anti-mouse immunoglobulin G–horseradish peroxidase (1:5000 dilution; Agrisera, AS111772) and PBS-T. ECL detection was performed using WesternBright Quantum (K-12042-D10, Advansta) Western blotting substrate and Amersham ImageQuant 800 imaging system (Cytiva).

### Structural modeling

The structures of tRNA^Gly1^-bound FaRel2, PhRel2, and CapRel^SJ46^ in the ATP-liganded state were predicted using AF3 (*30*) via AlphaFold Server (https://alphafoldserver.com/).
