# Supplementary material for: Toxic small alarmone synthetase FaRel2 inhibits translation by pyrophosphorylating tRNAGly and tRNAThr
Source: Sci Adv. 2024 Nov 13;10(46):eadr9624. doi: 10.1126/sciadv.adr9624 (PMC11559606; doi:10.1126/sciadv.adr9624)
Supplement: Supplementary file 1 — Figs. S1 to S7 Legends for tables S1 to S3 [file sciadv.adr9624_sm.pdf]

Science Advances
AAAS

Supplementary Materials for

Toxic small alarmone synthetase FaRel2 inhibits translation by pyrophosphorylating tRNA^{Gly} and tRNAThr

Tatsuaki Kurata *et al.*

Corresponding author: Abel Garcia-Pino, abel.garcia.pino@ulb.be; Tsutomu Suzuki, ts@chembio.t.u-tokyo.ac.jp; Vasili Hauryliuk, vasili.hauryliuk@med.lu.se

Sci. Adv. **10**, eadr9624 (2024)
DOI: 10.1126/sciadv.adr9624

The PDF file includes:

Figs. S1 to S7
Legends for tables S1 to S3

Other Supplementary Material for this manuscript includes the following:

Tables S1 to S3

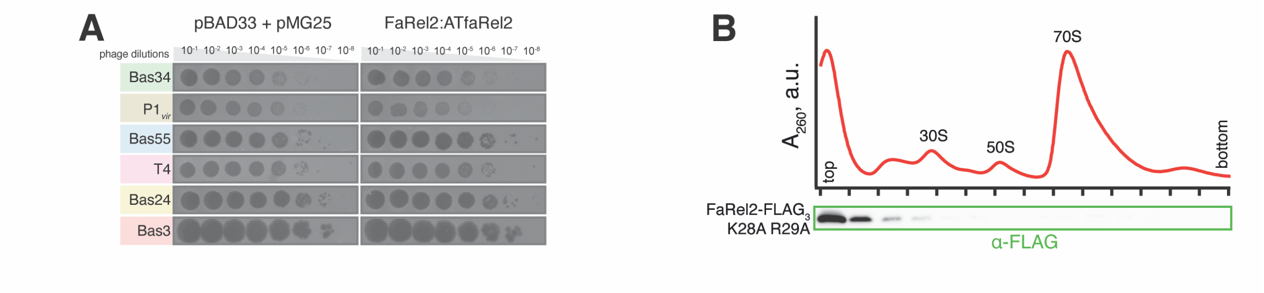

fig. S1. Functional studies of *Coprobacillus* sp. D7 FaRel2:ATfaRel2 TA system.

(**A**) *Coprobacillus* sp. D7 FaRel2:ATfaRel2 TA does not afford protection against coliphages when heterologously expressed *E. coli*. Serial dilutions of select BASEL (*28*) and common laboratory phages were spotted on a lawn of BW25113 *E. coli*, either expressing FaRel2:ATfaRel2 TA system or transformed with empty pBAD33 and pMG25 plasmids. Experiments with representative phages (out of >60 tested) are shown. (**B**) FaRel2 does not stably associate with ribosomes. A lysate prepared from *E. coli* cells expressing a non-toxic FaRel2 variant (FaRel2-FLAG$_3$ K28A R29A) was fractionated on a 10-35% sucrose gradient, and the FLAG$_3$-tagged FaRel2 K28A R29A protein was detected by Western blotting with anti-FLAG antibodies.

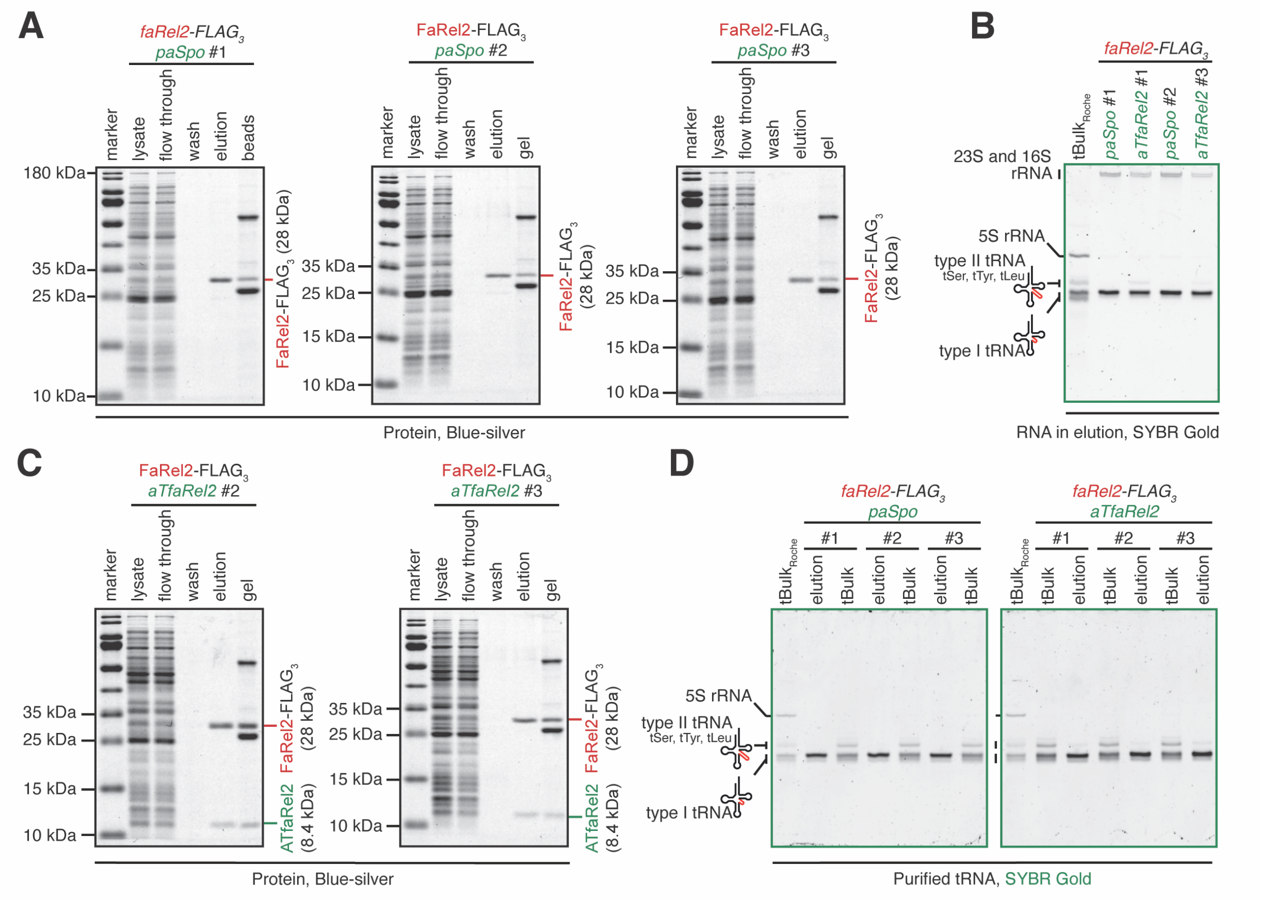

fig. S2. Immunoprecipitation of FaRel2-FLAG3 and FaRel2-FLAG3:ATfaRel2, two replicates, related to Fig. 2.

(**A**) Three biological replicates of a-FLAG3 immunoprecipitation of FaRel2-FLAG3. (**B**) Two additional biological replicates of RNA co-eluted with either FaRel2-FLAG3 or FaRel2-FLAG3:ATfaRel2 compared to $tBulk_{Roche}$. (**C**) Two replicates of anti-FLAG3 immunoprecipitation of FaRel2-FLAG3:ATfaRel2. (**D**) Final tRNA fractions used for mim-tRNAseq. $tBulk_{Roche}$ stands for commercial preparation of *E. coli* small RNA fraction, while tBulk designates lab-made preparations.

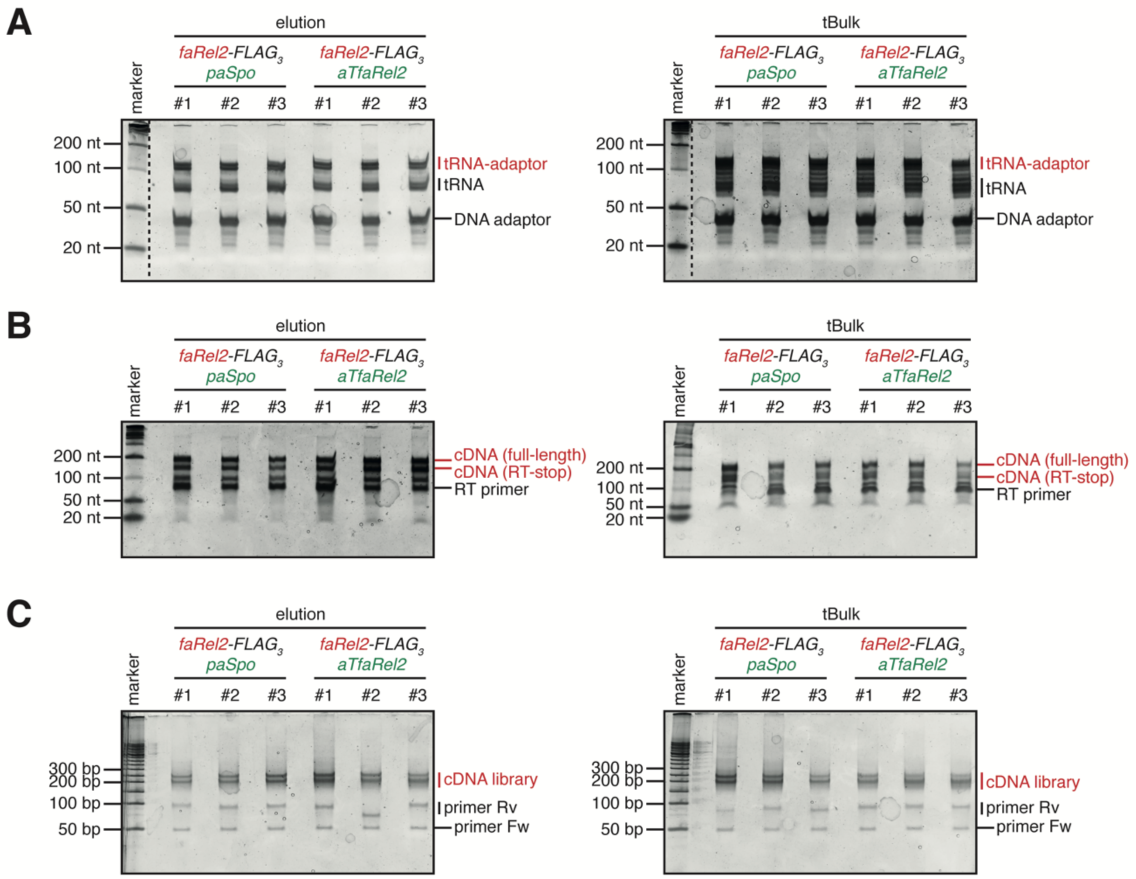

fig. S3. Gel images of samples in the preparation for mim-tRNAseq.

Three biological replicates of tBulk preparations and FaRel2-bound tRNA preparations after adaptor ligation (**A**), reverse transcription (**B**), and PCR reaction for cDNA library construction (**C**) were resolved on urea-PAGE in 1x TBE (7 M urea, 10% PAGE) or native-PAGE (6% PAGE) in 1x TBE, stained with SYBR Gold, and subjected to gel purification.

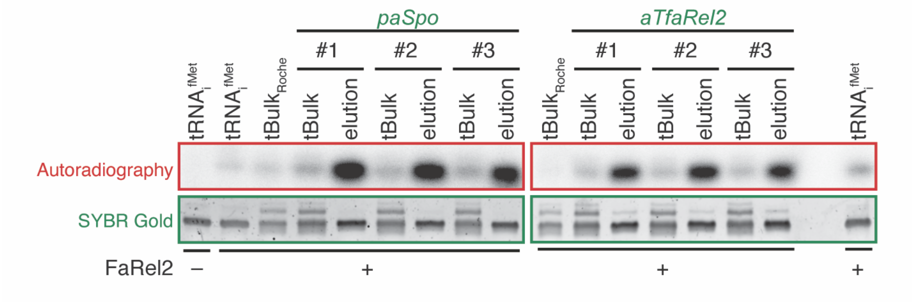

fig. S4. tRNA fraction associated with FaRel2-FLAG$_3$:ATfaRel2 is efficiently modified by FaRel2, related to Fig. 4.

Pyrophosphorylation assays using ^{32}P-labelled ATP and unlabelled tRNA substrates. FaRel2-FLAG$_3$- or -FLAG$_3$:ATfaRel2-coimmunoprecipitated tRNA fractions are more efficiently modified FaRel2 as compared to tBulk and individual *E. coli* tRNA$_i^{fMet}$. 100 nM FaRel2-FLAG$_3$ was reacted with 0.4 µM tRNA substrates at 37°C for 10 min. tBulk$_{Roche}$ stands for commercial preparation of *E. coli* small RNA fraction, while tBulk designates lab-made preparations.

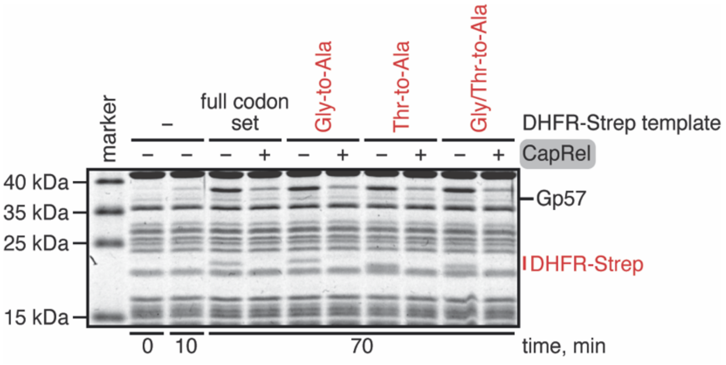

fig. S5. tRNA specificity of CapRel^{SJ46} is different from that of FaRel2.

SECΦ27 major capsid protein Gp57, the trigger of CapRel^{SJ46} toxSAS, was produced *in situ* from the template plasmid (10 ng/μl) in the PURE cell-free protein synthesis system. Gp57 was synthesised either in the presence or absence of purified CapRel^{SJ46} (250 nM). Next, the DHFR template plasmids were added (20 ng/μL) and the DHFR reporter proteins were synthesised for 60 minutes at 37°C. The reporters used: i) the full codon set version that encodes all of the possible codons, ii) Gly-to-Ala variant in which all Gly codons substituted for Ala, iii) Thr-to-Ala, all Thr codons substituted for Ala, iv) Gly/Thr-to-Ala, all Gly and Thr codons substituted for Ala. Addition of CapRel^{SJ46} abrogated the production of all of the tested Strep-tagged DHFR reporters. The experiment was performed once.

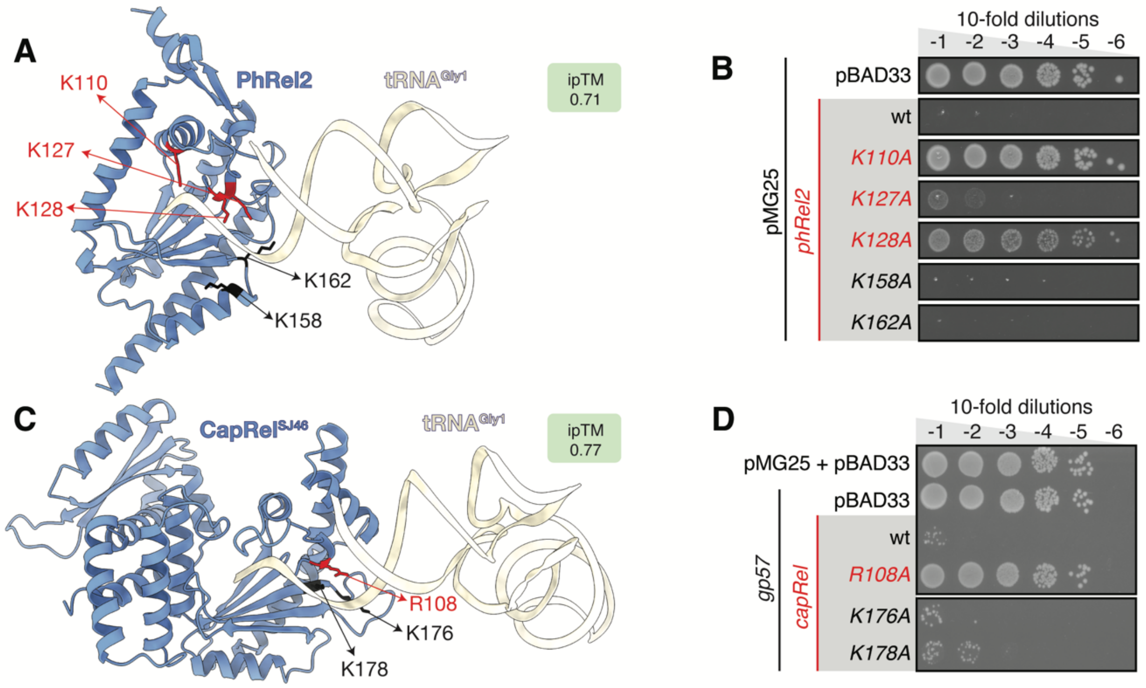

fig. S6. AF3-generated structures of tRNA^{Gly}-bound *B. subtilis* la1a PhRel2 and CapRel^{SJ46} and their mutational probing.

(**A,B**) AF3-predicted structure of *B. subtilis* la1a PhRel2 in complex with *E. coli* tRNA^{Gly} (A) and its mutational validation in toxicity assays (B). (**C,D**) Predicted structure of CapRel^{SJ46} in complex with *E. coli* tRNA^{Gly} (C) and mutational validation in toxicity assays (D). (B,D) Ten-fold dilutions of overnight cultures of *E. coli* strains transformed with pBAD33 vector or pBAD33 derivatives expressing either wild-type or mutant toxSAS variants and either pMG25 vector or pMG25 derivatives expressing Gp57 were spotted on LB plates and scored after a 16-hour-long incubation at 37°C. The experiments were performed two times, representative plates are shown.

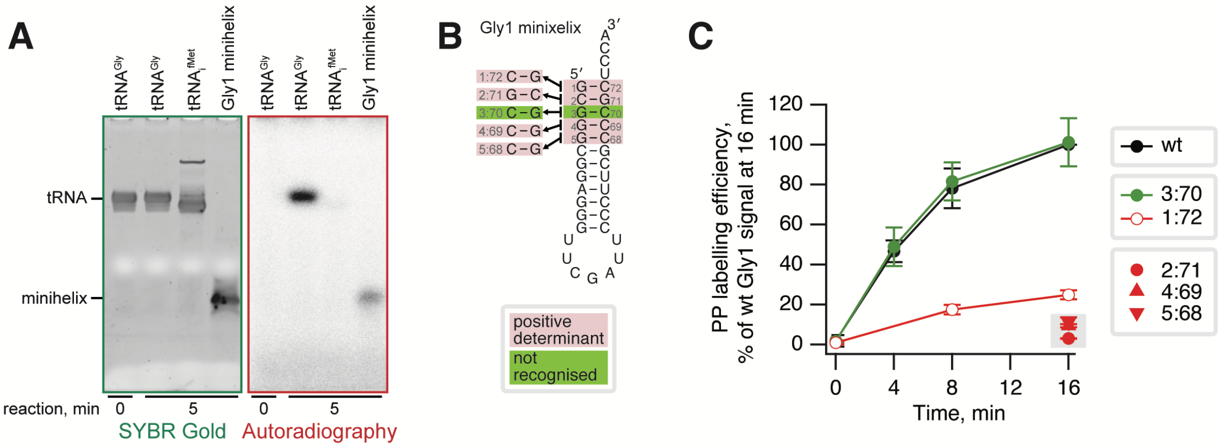

fig. S7. Validation of the tRNA^{Gly1}-mimicking RNA minihelix as an experimental model and kinetic analysis of Gly1 minihelix (wild type and mutant variants) modification by FaRel2. (**A**) Pyrophosphorylation of native *E. coli* tRNAs as well as synthetic tRNA^{Gly1}-mimicking RNA minihelix by FaRel2. The reaction mixture containing 5 µM RNA substrates, 100 µM ^{32}P-labelled ATP and 5 nM FaRel2-FLAG$_3$ was incubated at 37°C for 5 minutes and then quenched with RNA dye. The samples were resolved on 12% urea-PAGE and visualized by SYBR Gold staining as well as by autoradiography. The experiment was performed twice, a representative gel is shown. (**B**) Predicted secondary structure of tRNA^{Gly1}-mimicking RNA minihelix as well as mutations used in kinetic experiments. (**C**) Time course of FaRel2-mediated pyrophosphorylation of synthetic Gly1 RNA minihelix substrates. 5 µM substrates were modified at 37°C for 4, 8 or 16 minutes by 10 nM FaRel2-FLAG$_3$ in the presence of 100 µM ^{32}P-labelled ATP, and then the reactions were quenched, RNAs resolved on 12% urea-PAGE and visualized using SYBR Gold staining and autoradiography. The autoradiography signal was normalized to SYBR Gold signal, and expressed as a faction of the signal intensity for wild-type Gly1 substrate. Signal intensity for wild-type Gly1 at 16 minutes was set to 100 %. The experiment was performed in triplicates and the quantified data is shown as average ± standard deviation.

table S1. (separate file)

min-tRNAseq read counts.

table S2. (separate file)

Acceptor stem and complete sequences of *Coprobacillus* sp. D7 $tRNA^{Gly}$, $tRNA^{Thr}$ and $tRNA^{Leu}$ tRNA species as well as *E. coli* BW25113 complete tRNA sequences.

table S3. (separate file)

Strains, plasmids and oligonucleotide primers used in this study. Tables are in individual tabs with the following information: plasmids, cloning procedures, primers and strains.
